# Evaluation of a Library of FDA-Approved Drugs for Their Ability To Potentiate Antibiotics against Multidrug-Resistant Gram-Negative Pathogens

**DOI:** 10.1128/AAC.00769-19

**Published:** 2019-07-25

**Authors:** Charlotte K. Hind, Christopher G. Dowson, J. Mark Sutton, Thomas Jackson, Melanie Clifford, R. Colin Garner, Lloyd Czaplewski

**Affiliations:** a Research and Development Institute, National Infection Service, Public Health England, Porton Down, United Kingdom; b Life Sciences, University of Warwick, Coventry, United Kingdom; c Radcliffe Department of Medicine, MRC Molecular Haematology Unit, MRC Weatherall Institute of Molecular Medicine, University of Oxford, Oxford, United Kingdom; d Antibiotic Research UK, York, United Kingdom; e Chemical Biology Ventures Ltd., Abingdon, United Kingdom

**Keywords:** repurposing, antibiotic resistance breakers, antimicrobial combinations

## Abstract

The Prestwick library was screened for antibacterial activity or “antibiotic resistance breaker” (ARB) potential against four species of Gram-negative pathogens. Discounting known antibacterials, the screen identified very few ARB hits, which were strain/drug specific. These ARB hits included antimetabolites (zidovudine, floxuridine, didanosine, and gemcitabine), anthracyclines (daunorubicin, mitoxantrone, and epirubicin), and psychoactive drugs (gabapentin, fluspirilene, and oxethazaine). These findings suggest that there are few approved drugs that could be directly repositioned as adjunct antibacterials, and these will need robust testing to validate efficacy.

## Abstract (precis)

The Prestwick library was screened for antibacterial activity or “antibiotic resistance breaker” (ARB) potential against four species of Gram-negative pathogens. Discounting known antibacterials, the screen identified very few ARB hits, which were strain/drug specific.

## TEXT

The need for new antibiotics is driven by the rapid spread of multidrug-resistant (MDR) bacterial pathogens, and the absence of new antibiotics in the clinical development pathway is significant cause for concern. The idea of repurposing existing drugs that are currently being used as treatments for other diseases is attractive because, due to the known safety profiles of approved drugs, the cost and time to the clinic could be significantly less than for novel scaffolds (1). Successful repurposing screens, outside of the antibacterial area, have produced candidates for Ebola virus, Zika virus, and anticancer therapies (2–4). Recent studies for the identification of new antibacterial leads have focused on two key areas, i.e., (i) identification of direct antibacterial hits for one or more target bacteria (5, 6) and (ii) screening for compounds that synergize with existing antibiotics, thereby restoring activity of the antibiotic against strains/species that are currently resistant to their use (7). Several previous studies identified antibacterial activities that are too weak to be effective on their own and would require exposures greater than the maximum concentration achievable with their primary pharmacology and recommended safe dosing (7), possibly because of the bacterial membrane barriers.

The current study aimed to identify either direct-acting antibiotics or compounds that sensitize resistant Gram-negative strains to one or more antibiotics, looking to identify “antibiotic resistance breakers” (ARBs). A high-throughput combination screen (HTCS) of potential ARBs and antibiotics was performed in a 384-well format, from the Prestwick library of 1,280 selected compounds in combination with 5 antibiotics or 0.1% dimethyl sulfoxide (DMSO), in duplicate. Each replicate was from independent dilution plates, using independent inocula on different days. The potential ARBs were tested at two concentrations, 20 μM and 7 μM, in combination with antibiotics at 0.125× MIC. Concentrations were selected to balance the probability of achieving a significant number of hits with realistic concentrations that aligned with the likely maximum concentration for a typical drug. When the MIC was >128 mg/liter, the antibiotic was tested at 16 mg/liter. The MICs of test articles were determined in cation-adjusted Mueller-Hinton broth (Oxoid), according to Clinical and Laboratory Standards Institute (CLSI) guidelines (8, 9).

Clinical isolates of Escherichia coli, Pseudomonas aeruginosa, Klebsiella pneumoniae, and Acinetobacter baumannii (which were recently highlighted by the World Health Organization as priority pathogens for which new antibiotics are urgently required [10]) that were resistant to each antibiotic were selected. For some species (K. pneumoniae and A. baumannii), this involved the use of two strains to cover all resistance profiles, and some resistance profiles were not available (see Table S1 in the supplemental material).

During the HTCS, bacterial growth was determined by reading at 600 nm, on a modal reader (Infinite 500; Tecan), after 24 h of incubation. For each plate, measurements of the optical density at 600 nm (OD_600_) were made at two time points, i.e., at 0 h (to determine the background signal related to the colored compounds) and at 24 h (at the end of the incubation). After blank substitution, calculated by subtracting the OD_600_ at 0 h from the OD_600_ at 24 h, a normalization step was carried out for OD_600_ values obtained in wells containing the compounds, compared with values obtained in control wells (DMSO wells – maximal growth). Data analysis for each run was performed with Genedata Screener software. The workflow from the raw data associated with the plate map up to the normalization step was fully automated, allowing complete tracking of all data. The Z′ factor and assay window were determined for each plate, between the positive control in the presence of antibiotic at 0.125× MIC and the negative control (11). The Z′ factor for each combination of strain and antibiotic was between 0.5 and 0.8, and plates displaying Z′ factors of <0.5 were automatically retested.

After statistical analysis, hits were defined as data points with activities greater than the hit threshold, based on the sigma method (mean + 3 standard deviations), unless otherwise stated. Results were expressed as percent growth inhibition, compared to the growth of untreated controls (exposed to 0.1% DMSO only), as assessed by optical density.

Firstly, compounds from the library were tested for direct antimicrobial activity at two concentrations, 7 μM and 20 μM, in the presence of 0.1% DMSO (Fig. S1 and S2). The number of direct hits at either concentration varied considerably between species, with 29 hits for E. coli, 16 hits for P. aeruginosa, 85 hits for the two A. baumannii strains combined, and 53 hits for the two K. pneumoniae strains (discounting overlapping hits between the two strains of the same species and between the two concentrations tested) (Table S2). As might be expected, we saw three scenarios with respect to dose-response relationships, i.e., (i) compounds that were equally effective at the two concentrations, (ii) compounds that were effective at 20 μM but were not effective as either direct antibacterials or ARBs at 7 μM, and (iii) compounds that were ARBs at 7 μM but were directly antibacterial at 20 μM.

**FIG 1:**
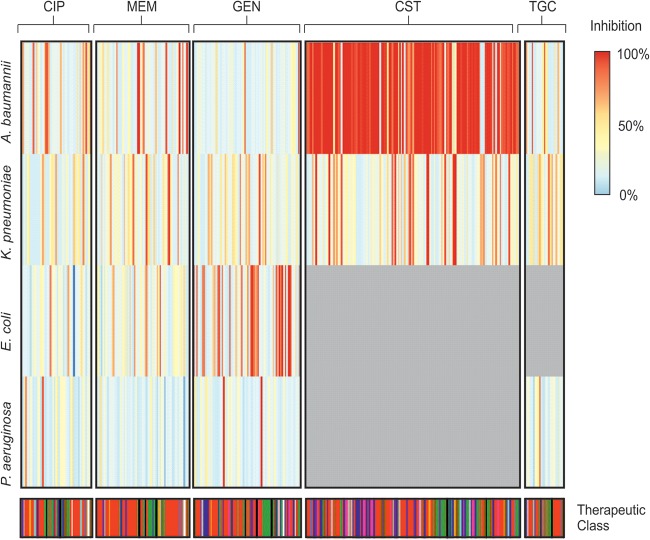
Heat map showing ARB hits by species and antibiotic potentiated. The heat map is colored according to the amount of growth inhibition caused in each species in combination with each antibiotic (gray indicates that the combination was not tested). Few ARB hits show any conservation across species or with specific antibiotics. CIP, ciprofloxacin; MEM, meropenem; GEN, gentamicin; CST, colistin; TGC, tigecycline.

Compounds at 7 μM or 20 μM were also tested in combination with antibiotics at concentrations of 0.125× MIC. There were few hits that overlapped between species (Fig. 1). Most of the compounds that did overlap were known antimicrobials or antiseptics (Tables S5 to S10). A number of compounds showed interesting potentiation; these are discussed below and in the supplemental material.

Three anthracycline-related molecules, namely, daunorubicin, mitoxantrone, and epirubicin, showed potentiation with one or more combinations of drug and species (Table 1). The pattern of activity differed between the three molecules tested, with no evidence of direct antibacterial activity but differing levels of potentiation for other antibiotics.

**TABLE 1:**
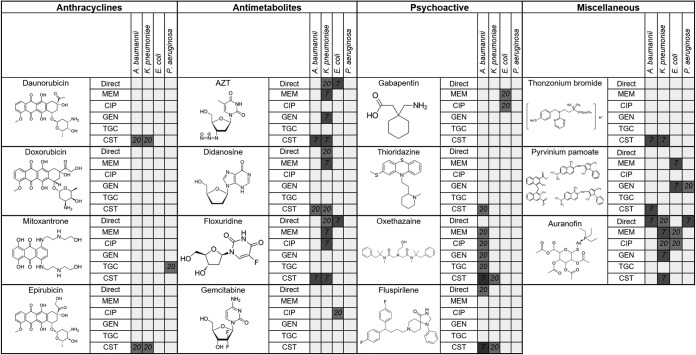
Structures and antimicrobial profiles of interesting hits from the screen^*a*^

a Shaded boxes illustrate direct or ARB activities (in micromolar) of compounds in combination with meropenem (MEM), ciprofloxacin (CIP), gentamicin (GEN), tigecycline (TGC), or colistin (CST) in the four Gram-negative species tested. For compounds that had activity at both 20 μM and 7 μM, only 7 μM is represented in the table.

Several nucleotide/nucleoside analogues, identified as antimetabolites and/or antiviral agents, also showed potentiation with one or more antibiotic (Table 1). While simplistically such molecules might be expected to have similar effects via interference with DNA/RNA metabolism in the cell, there were clear differences in the spectra of activity of the compounds.

Two psychoactive compounds, fluspirilene and oxethazaine, were also found to act as ARBs with colistin and merited further investigation, given the possibility that their mode of action might be different from that of cationic compounds identified previously as being able to potentiate colistin (for example, pentamidine [12], which was not found to potentiate colistin activity in this study, and cysteamine, which was not included in this study [13]). The MICs of colistin, alone and in combination with set concentrations of fluspirilene and oxethazaine, were determined as described above but using non-cation-adjusted Mueller-Hinton broth (Oxoid) and polypropylene plates, with incubation for 20 h at 37°C (14).

Colistin potentiation by fluspirilene and oxethazaine in a larger panel of colistin-resistant strains of K. pneumoniae and a smaller number of other Gram-negative pathogens was tested as an example of compounds that were clear ARBs with very little direct antimicrobial activity (Table S3). The studies were designed as fixed concentration-synergy experiments, looking for ARB activity. Initially, MICs and growth curves were used to analyze the direct effects of the two compounds. In most cases, the MICs were >160 μM for *Klebsiella* sp. and P. aeruginosa isolates. For E. coli, all strains had MICs of 160 μM or above for oxethazaine, but two strains (LEC001 and 319238/UR) had MICs of 80 μM for fluspirilene. The notable exceptions to the high MIC values identified were the A. baumannii strains, which showed MICs of 20 μM for both oxethazaine and fluspirilene in both colistin-resistant strains (Table S4).

**FIG 2:**
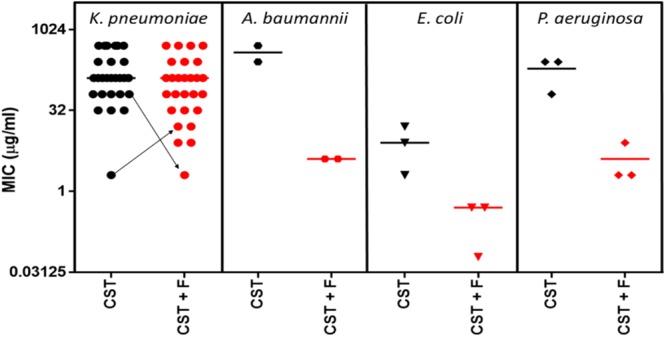
Colistin ARB potential of fluspirilene. A larger panel of colistin-resistant strains was tested in the presence of fluspirilene. Although the K. pneumoniae strain used in the HTCS showed colistin potentiation by fluspirilene, this was not reflected in the larger panel. However, fluspirilene did potentiate colistin in other Gram-negative species. Arrows in the K. pneumoniae panel indicate the changes in MICs for two specific strains. This represents an example in which fluspirilene is antagonistic to colistin but the MIC is in the same range as in some strains where potentiation is observed. CST, colistin; F, fluspirilene.

Despite being ARB hits with the original colistin-resistant K. pneumoniae strain used in the HTCS, there were few examples of clear colistin potentiation with either compound within the larger panel of *Klebsiella* isolates. Only strains NCTC 13439 CST 2A (4-fold), MGH 78578 CST A (8-fold), and m109 CST 1B (32-fold) showed >2-fold potentiation of colistin with fluspirilene (Fig. 2; also see Table S3), and no strains showed this level of potentiation with oxethazaine.

In contrast, fluspirilene showed potentiation of colistin with all of the other Gram-negative species tested, with levels ranging from 4-fold (A. baumannii W1 CST_R) to >128-fold (E. coli LEC001). The latter strain was also the only strain that showed potentiation with oxethazaine, again with >128-fold increased susceptibility to colistin. Whether derivatives of fluspirilene merit further investigation as standalone antibiotics or as ARBs may depend on the novelty of the mechanism of action. The developability is hampered by the relatively high concentration required to achieve potentiation of colistin (for example, around 20 μM [equivalent to 9.5 mg/liter] against K. pneumoniae), compared to the daily dose (10 mg per day, intramuscularly).

The current screen, in line with many other studies, suggests that there might be very few licensed drug compounds that could simply be repositioned and have immediate benefit as adjunct therapies. This finding does not preclude future studies looking at other antimicrobial strategies, such as biofilm disruption (5), antivirulence activity (15), or efflux pump inhibition (16), but it does suggest that such studies must be carefully designed to generate useful information. The screening of existing approved drugs, while attractive from a regulatory standpoint and as rapid route to market, does not directly address the challenges of antimicrobial drug development, including the permeability issue, which affects drug uptake into Gram-negative bacteria (17), or the relatively limited chemical space inhabited by most classical drugs (18).

## Supplementary Material

Supplemental file 1

Supplemental file 2

Supplemental file 3

Supplemental file 4

Supplemental file 5

Supplemental file 6

Supplemental file 7
