# Supplementary material for: Evaluation of a Library of FDA-Approved Drugs for Their Ability To Potentiate Antibiotics against Multidrug-Resistant Gram-Negative Pathogens
Source: Antimicrob Agents Chemother. 2019 Jul 25;63(8):e00769-19. doi: 10.1128/AAC.00769-19 (PMC6658780; doi:10.1128/AAC.00769-19)
Supplement: Supplemental file 1 [file AAC.00769-19-s0001.pdf]

Supplementary results and discussion.

The observed hit rate per species of between 1.25 – 6.6 % is considerably higher than might be expected from a screen such as this, most likely due to the make-up of the Prestwick library, which includes a number of known antimicrobial agents, antiseptics and other anti-infectives. Indeed between 72.5 % (*E. coli*) and 93.8 % (*P. aeruginosa*) of the hits were known antimicrobials (antibacterial and antifungal) or antiseptics, while the remainder were from other therapeutic classes. In similar screens run for direct antibacterial activity, using the same library of compounds, other studies have shown a hit rate of 3.25% against *Candida albicans* and 2.65% against *P. aeruginosa* for individual strains / species [2, 3].

Clinical isolates were selected which were resistant to antibiotics representative of 5 antimicrobial classes with different modes of action; carbapenems (meropenem, MEM), fluoroquinolones (ciprofloxacin, CIP), aminoglycosides (gentamicin, GEN), tetracyclines (tigecycline, TGC) and polymixins (colistin, CST) (Table S1). In the case of *K. pneumoniae* and *A. baumannii*, 2 strains were used which between them had resistance to all 5 classes of antibiotics. No CST-resistant *P. aeruginosa* or TGC-resistant *E. coli* strains were available, and resistance to CST in the *E. coli* strain ATCC BAA-2469, which contains the *mcr-1* plasmid, was only just above the breakpoint and not reliable (MIC 1 – 2 mg/L). As such, CST-resistant *E. coli* and *P. aeruginosa* and TGC-resistant *E. coli* were not available for the initial screening phase.

For ARBs the corrected percent inhibition was defined by growth inhibition in the presence of compound plus antibiotic minus the growth inhibition in the presence of compound plus DMSO control. The maximum inhibition was then defined as the highest corrected percentage inhibition observed between the two duplicates of the HTS. Finally, ARB hits were defined as maximum corrected percent inhibitions that were greater than the mean + 3SD of the maximum inhibitions (Figure 2 and supplementary figures 2-6). False positive ARBs, particularly from wells where the use of DMSO alone gave an aberrantly high growth inhibition which masked any ARB activity, were included in the initial analysis, but haven't been discussed further in this manuscript.

There are some striking differences with the numbers of ARB hits between bacterial species (Table S2). The highest number of ARB hits was found in combination with CST, which is most likely due to the ability of CST to permeabilise the outer membrane of select Gram-negative species, thereby effectively becoming the ARB for the test compounds and allowing them into the cell to interact with an intracellular target [4]. The majority of hits for *K. pneumoniae* were known antimicrobials or antiseptics, whereas for other species, up to half of the hits were compounds with no previously described antimicrobial activity. Interestingly, for combinations of compounds with GEN, the highest number of ARB hits was found in *E. coli* (20 ARBs), which had ten times the amount of hits identified for *K. pneumoniae* (2 ARBs). Very few hits were observed in *P. aeruginosa* with any of the compounds directly (16 hits) nor in combination with antibiotics (40 ARBs across all antibiotic combinations). Aside from the high number of ARB hits identified with CST, hits were distributed by antibiotic with the following frequency, irrespective of species (GEN (107) > MEM (81) > CIP (75)).

There were interesting observations around potentiation of different antibiotic classes by a number of antiseptics and interactions between existing antimicrobials (mostly weak antibacterials which had greater potency when combined with CST, such as rifampicin, furazolidone, clioquinol and nitrofurantoin). The strongest combination of known antimicrobials, CST and rifampicin, has known

synergistic interactions *in vitro*, which has so far failed to translate *in vivo* [5]. As these were not the focus of the current study, these have not been discussed further. The focus of this study was to identify new compounds lacking known or demonstrable antimicrobial effects which might work synergistically with one or more antibiotic class. The intention would be to look at these as candidates for direct repositioning as adjunct therapies. Amongst the hits identified, it was possible to identify three groups of compounds that showed potentiation in more than one member of the class (Table 1). However, most are cytotoxic and future studies will need to carefully consider whether there is a therapeutic window which would support development of such molecules as adjunct therapies.

Anthracyclines and derivatives.

The anthracyclines are 4-ring heterocyclic compounds originally identified from natural products derived from *Streptomyces* and related species. They are some of the most widely used drugs for chemotherapy for a variety of different tumour types. Within this class, the clearest potentiation in this screen was with daunorubicin/epirubicin and CST, against both *K. pneumoniae* and *A. baumannii,* with >93% reduction in growth observed in both species at 20 μM. ARB activity with mitoxantrone was limited to potentiation of TGC against *P. aeruginosa* in this study, but the literature has reported synergy with imipenem in *S. aureus* and *E. coli* and with CST in the laboratory strain PA01 [3, 6].

The mechanism of action of anthracyclines is multifactorial, but key to their efficacy as chemotherapy agents is their ability to intercalate between bases in DNA or RNA and poison topoisomerase II, leading to toxic double stranded DNA breaks and causing cell death. The observed activity in bacteria is likely to be via a similar mechanism. Whilst this likely conservation of mechanism may preclude clinical development as an adjunct antimicrobial therapy, encapsulation of anthracyclines to minimise off-target effects and targeting to increase specificity might offer possible routes forward. Such approaches have been widely explored for improving the target specificity and reducing the general toxicity for chemotherapy drugs [7, 8]. Interestingly the related anthracycline compound, doxorubicin, was also included in the screen but showed no activity, either as a direct hit or an ARB. This perhaps suggests that the mechanism of potentiation is not simply related to toxicity of the compounds, but is mediated by a specific cellular mechanism. Anthracycline analogues with an improved therapeutic index as antibacterials have also been identified, suggesting that there may be merit in exploring this class of molecule further [9]. Strategies to explore other DNA binding molecules, currently being used as chemotherapy agents, have also been explored with pyrollobenzodiazepines being the subject of recent papers [10, 11] (Picconi, Hind *et al.* manuscript in preparation).

Antimetabolites.

Zidovudine (ZDV), also known as azidothymidine (AZT), is a thymidine analogue that inhibits the reverse transcriptase of HIV [12]. The compound showed direct antibacterial activity against *K. pneumoniae* at 20 μM and *E. coli* at 7 μM. ARB effects are also seen with MEM, GEN and CST in *K. pneumoniae* and in *A. baumannii* with CST only. Direct activity of ZDV is well characterised in *E. coli* [13], where ZDV appears to have dual targets, thymidine phosphorylase [14] and thymidine kinase [15]. ZDV requires activation to its triphosphate version by cellular enzymes and, whilst this may improve its safety profile, the rate of this activation mechanism in different species may contribute to

differential activity between species. ARB activity has also been reported previously, most notably with CST in *Enterobacteriaceae* [16, 17]. There are also reports of ZDV working synergistically with trimethoprim in *Enterobacteriaceae* with the effects being enhanced by inclusion of floxouridine, perhaps by modulating intracellular nucleotide/nucleoside pools [18].

Floxuridine (5-fluorodeoxyuridine), a pyrimidine analog used in cancer therapy, showed direct activity against *E. coli* at 7 μM and *K. pneumoniae* at 20 μM and significant potentiation of MEM, CIP and CST in *K. pneumoniae* and CST in *A. baumannii* at 7 μM. Activity of floxuridine has been reported previously, also against *E. coli* and *K. pneumoniae*, but was shown to be bacteriostatic (unlike ZDV which was rapidly bactericidal) [19]. The study highlighted the potentially poor prospects of developing the drug as an antimicrobial, given its high toxicity, low recommended dose of 0.3 mg/kg daily [20] and very short plasma half-life of around 15 minutes [21]. This is perhaps exacerbated by common side effects, seen in around 30% of patients, which include low blood counts (platelets and white cells) and diarrhoea [22].

The third antimetabolite compound, didanosine, a nucleoside analogue of adenosine used as an antiviral, showed ARB activity with MEM at 7 μM and CST at 20 μM against *K. pneumoniae* and CST against *A. baumannii* at 20 μM . Previous studies have reported direct antibacterial activity of didanosine against *E. coli* and *Salmonella*, but this was relatively modest (31 - 62 and 2 – 125 mg/L respectively) which was reflected here in *K. pneumoniae* with direct activity of just 30 % growth inhibition at 20 μM. Previous ARB effects have not been reported to our knowledge. Opportunities to further develop this compound as an adjunct therapy may be limited by the low Cmax (~2 mg/L) described at normal doses while didanosine might require significant dose escalation to achieve relevant therapeutic effects [23].

A fourth antimetabolite drug, gemcitabine, showed limited but intriguing potentiation with CIP in *E. coli* at 20 μM. Gemcitabine is another nucleoside analogue used to treat patients with a variety of different cancers. Recently there has been considerable interest in the relationship between the microbiome and gemcitabine, notably the suggestion that intratumour bacteria are capable of inactivating the drug via the action of cytidine deaminase enzymes, generating the inactive form 2',2'-difluorodeoxyuridine [24]. The activity is a function of the long form of the bacterial cytidine deaminase enzyme, which is expressed in *E. coli* along with many other Proteobacteria. The use of prodrug versions of floxuridine and gemcitabine similar to those generated for ZDV [25], might be a possible route forward, as might the generation of amino-acid conjugated prodrugs with improved safety profiles [26], and targeting bacterial uptake.

Other nucleoside analogues, abacavir, emtricitabine, lamivudine, stavudine and zalcitabine were included in the library but showed no direct or ARB activity.

Psychoactive drugs.

Gabapentin, an analogue of the neurotransmitter gamma-aminobutyric acid (GABA), used to treat neuropathic pain, was an ARB at 20 μM in *E. coli* with MEM and CIP. Gabapentin is already used to treat side effects of diarrhoea infections caused by *E. coli* and the ARB activity may be a useful component of this therapy. Gabapentin has a Cmax of 8.7 mg/L which is compared to a concentration of 3.4 mg/L at 20 μM. Previous studies have suggested direct antibacterial activity against *S. aureus, P. aeruginosa and E. coli* [27], but that was not observed in other studies [28]. The

latter study identified a number of other psychoactive compounds with broad ranging antibacterial activity including sertraline (not a hit or ARB in this study) and fluoxetine (ARB with CST in *A. baumannii*). Thioridazine, an antipsychotic phenothiazine drug, also showed ARB activity with CST in *A. baumannii* at 20 μM. Interestingly, thioridazine has been shown to have ARB properties in studies with oxacillin and dicloxacillin in MRSA [29, 30] as well as direct antimicrobial effects against TB [31]. We have also previously shown that fluoxetine and thioridazine can impact on biofilm formation in *Proteus mirabilis*, with a mechanism of action that relates to inhibition of a specific MFS-family efflux pump [32].

Fluspirilene, is a diphenylbutylpiperidine drug used in the treatment of schizophrenia and anxiety disorders, working via blockade of the dopamine D2 receptors. The compound showed potentiation of CST with both *A. baumannii* (7 μM) and with *K. pneumoniae* (20 μM) as well as direct antibacterial activity against *A. baumannii* at 20 μM. This data largely confirms the results from a recent publication, which also described ARB activity in *A. baumannii* with both CST and azithromycin [33]. Interestingly, fluspiriline has also been explored to identify new drug targets via target-independent hit expansion and target identification, suggesting this scaffold may have general properties that are useful for repurposing [34].

Oxethazaine is used as a local anaesthetic and a component of treatments for ulcers, and works by altering the sensitivity of the membranes of gastric or sensory cells to sodium [35]. It acted as an ARB with CST at 7 μM in *K. pneumoniae* and 20 μM in *A. baumannii*. There are no previous descriptions of activity of oxethazaine as either a direct antibacterial or as an ARB.

A more in-depth exploration of the ARB activities of fluspirilene and oxethazaine in combination with colistin was conducted in a larger panel of colistin-resistant strains. When MICs of fluspirilene and oxethazaine were assessed as growth curves, a number of additional strains showed significant growth defects at sub-MIC concentrations. The highest concentration that did not reduce growth by more than 20% was selected for ARB studies. This is reflected in the lower concentrations of both compounds used for potentiation studies with 2/3 *P. aeruginosa* strains (5 µM) and with *A. baumannii* strains (5 µM oxethazaine; Table S4). The compounds were used at 10 or 20µM for all other strains tested. The reason for the strains dependence and/or heteroresistance to these compounds was not investigated further.

The CST resistance associated mutations were the only mutations identified by whole genome sequence analysis in these particular strains (Table S3). NCTC 13439 CST 2A is the only *K. pneumoniae* strain tested with a mutation in *pmrA* and as such, this could be directly linked to the colistin-potentiation by fluspirilene. With respect to the two additional strains which show potentiation with fluspirilene, there are other strains with mutations in the same genes (*pmrB* and *mgrB*) which do not show CST potentiation. As other CST-resistant mutations in the same strain backgrounds (MGH 78578 and m109) did not show CST potentiation, the data suggests that the response in all three strains is also not linked to specific strain backgrounds. This suggests that the activity of fluspiriline as an ARB for CST may be related to specific alleles in each of the genes associated with CST resistance.

Intriguingly, one strain of *K. pneumoniae* (141 CHX) showed a significant loss of sensitivity to CST in the presence of either oxethazaine or fluspirilene. This strain has an MIC of 2 mg/L for CST in the

absence of potentiator and had the lowest MIC of any strain tested. The strain has a mutation in *wbaP* (encoding a single amino acid change WbaP D445E), one of the glycosyltransferases involved in O antigen lipopolysaccharide and polysaccharide capsule synthesis in other Gram-negative species. Whilst this mutation appears to have only a minor effect on CST resistance in this strain, it is linked to modestly elevated chlorhexidine resistance (from 16 mg/L to 32 mg/L; unpublished). Why this relatively conservative amino acid change should generate such a strong antagonistic effect to CST activity is not clear.

Other miscellaneous ARB hits

Thonzonium bromide was identified as a potent ARB with CST in both *A. baumannii* and *K. pneumoniae* and has previously been identified as a CST ARB against *P. aeruginosa* [3]. Thonzonium bromide is a cationic surface-active compound, used to promote dispersion and penetration of cellular debris as part of various formulations, promoting tissue contact of the administered medication. Examination of the structure suggested the presence of a quaternary amine group, which might explain its function as an ARB. Interestingly it did not show direct antibacterial activity, which is distinct from other cationic antiseptics (chlorhexidine, alexidine) which showed both direct activity and broad-spectrum ARB function in this screen. Its efficacy as an antibacterial is unclear, but it has been tested as a nanoparticle formulation for *Streptococcus mutans* biofilms [36] and described as a potential new anthelmintic following a drug repurposing screen with nematodes [37].

Pyrvinium pamoate, a quinolone-derived cyanine dye with a quaternary ammonium centre, used for treatment of pinworms, was an ARB for various drug and species combinations. It was most effective in *E. coli* where it was an ARB for GEN and MEM at 7 μM, also being an effective ARB with CST in *A. baumannii* (7 μM) and one of the few compounds to show effects in *P. aeruginosa* where it weakly potentiated GEN at 20 μM. Previous studies have described direct activity against MRSA [38] and *C. albicans* [2] and synergy with azoles against yeasts [39].

Auranofin, an antirheumatic agent, displayed ARB activity with MEM and CIP in *E. coli* and *K. pneumoniae*, and GEN and CST in *K. pneumoniae*. It was also directly antimicrobial against *A. baumannii, K. pneumoniae* and *P. aeruginosa.* Auranofin, a gold containing compound with anti-inflammatory properties, has been widely described as either a direct-acting antimicrobial against *C. albicans* biofilms [6], as an antiparasitic in phase I clinical trials against MRSA [7] and *K. pneumoniae* [8] and against both planktonic and biofilm cultures of *P. aeruginosa* (PAO1) in combination with CST [3].

Conclusions.

Aspects of the design of the current study, particularly the screen for ARB activity with a range of MDR Gram-negative species, was novel and produced some possible starting points for future drug discovery. The study reiterated the potential value of nucleoside/nucleotide analogues, anthracycline derivatives and psychoactive drugs as potential series which might merit additional investigation. There have already been a number of studies looking at either individual members of these classes as synergising agents (e.g. ziduvidine [19]) or more systematically [33, 40, 41].

The study also highlighted the challenges of extrapolating from single species/strains used in HTS to general utility in the species of interest. While fluspirilene and oxethazaine showed clear ARB activity in the original screen, the CST-resistant *K. pneumoniae* isolate was not representative of the majority

of other strains tested in extended studies, with only three isolates showing ARB effects and only with fluspirilene. This certainly asks interesting questions about the allele-specific nature of potentiation and its possible mechanisms, but it also highlights the pitfalls of such potentiation studies which are frequently reported in the literature with only a single or small number of closely related strains. The ARB effects seen with fluspirilene in the other CST-resistant Gram-negative species are encouraging, particularly with *E. coli,* where the MIC is reduced to below the breakpoint, but are again based on a very small sample size.

Supplementary figures

Table S1: MICs (mg/L) and relevant resistance genes for each organism/antibiotic combination in the high throughput combination screen. S = MIC below EUCAST breakpoints and therefore not relevant in this screen. (MEM; meropenem, CIP; ciprofloxacin, GEN; gentamicin, TGC; tigecycline, CST; colistin) EUCAST breakpoints were accessed by the following link (http://www.eucast.org/clinical_breakpoints/; accessed 3 May 2019).

Organism	Strain	Source	Known resistance genes	MEM	CIP	GEN	TGC	CST
A. baumannii	NCTC-13301		OXA-23[₸], OXA-51-like[₸]	32	>128	>128	2	S
	WP-487			S	S	S	S	2
K. pneumoniae	NCTC-13439		VIM-1[₸], qnrS1[¥]	S	4	S	2	S
	KPC-119			8	S	64	S	8
E. coli	ATCC BAA-2469		NDM-1[₸]	4	>128	>128	S	S
P. aeruginosa	NCTC-13437	Outbreak	VIM-10[₸], VEB-1[₸], GyrA[¥]	128	64	128	32	S

[₸] carbapenem resistance, [¥] fluoroquinolone resistance

Table S2: Direct hits and ARB hits in combination with existing antibiotics against Gram-negative strains. Numbers in brackets are the number of direct or ARB hits without previously utilised antimicrobial or antiseptic properties. Dashes represent untested conditions where the strains were sensitive to the combination antibiotic.

**Organism**	**Strain**	**Direct**		**MEM**		**CIP**		**GEN**		**TGC**		**CST**	
		20μM	7μM	20μM	7μM	20μM	7μM	20μM	7μM	20μM	7μM	20μM	7μM
A. baumannii	NCTC-13301	23 (3)	17 (3)	13 (8)	12 (6)	14 (7)	6 (4)	9 (7)	6 (4)	11 (10)	4 (3)	-	-
	WP-487	79 (5)	52 (1)	-	-	-	-	-	-	-	-	123 (86)	68 (38)
K. pneumoniae	NCTC-13439	30 (2)	13 (3)	-	-	14 (2)	9 (5)	-	-	15 (0)	9 (4)	-	-
	KPC-119	34 (4)	23 (5)	21 (2)	12 (4)	-	-	19 (2)	11 (2)	-	-	31 (10)	40 (24)
E. coli	ATCC BAA-2469	28 (8)	18 (5)	22 (7)	18 (5)	13 (7)	15 (5)	29 (20)	24 (11)	-	-	-	-
P. aeruginosa	NCTC-13437	16 (1)	8 (1)	6 (3)	5 (3)	18 (7)	8 (5)	17 (12)	7 (4)	8 (5)	5 (4)	-	-

Table S3: Extended panels of CST resistant strains show that potentiation is strain dependent. The concentrations of oxethazaine (Oxe) and fluspirilene (Flus) used in this combination screen are stated in Table S4.

			CST MIC (mg/L)		
**Species**	**Strain name**	**mutation**	**CST**	**+ Oxe**	**+ Flus**
K. pneumoniae	16 CST-R	PhoQ T244N	128	64	64-128
K. pneumoniae	NCTC 13438 CST C	Transposon insertion mgrB	128	256	256
K. pneumoniae	NCTC 13438 CST D	Del in pmrB ΔT18-G20	128	64	64-128
K. pneumoniae	NCTC 13439 CST 1A	Unknown	128	64	128
K. pneumoniae	NCTC 13439 CST 1C	PmrB P95L	32	32	32
K. pneumoniae	NCTC 13439 CST 2A	PmrA G53C	64	32	16
K. pneumoniae	NCTC 13439 CST 2B	MgrB D29A	64	32	32
K. pneumoniae	NCTC 13443 CST 2B	PmrB Duplication V72-L87	128	128	64-128
K. pneumoniae	NCTC 13443 CST	Not known	64	32	64
K. pneumoniae	MGH 78578 CST A	PmrB T157P	64	32	8
K. pneumoniae	MGH 78578 CST B	MgrB Δt9	128	128	64
K. pneumoniae	m3 CST D	PhoQ L348Q	512	256	512
K. pneumoniae	m109 CST 1B	MgrB G37C	64	64	2
K. pneumoniae	m109 CST 2B	PmrB Duplication I77-L87	32	32	32
K. pneumoniae	NCTC 51851	MgrB Q30STOP	512	512	512
K. pneumoniae	NCTC 49856	MgrB Q30STOP	512	256	512
K. pneumoniae	NCTC 13368 CHX	PhoP Y98C	128	128	64-128
K. pneumoniae	MGH 78578 CHX	PhoQ L348Q	512	512	512
K. pneumoniae	m109 CHX	unknown	128	128	128
K. pneumoniae	m3 CHX	PhoP E82K	256	256	128
K. pneumoniae	NCTC 13439 CHX	unknown	256	128	128
K. pneumoniae	NCTC 13443 CHX	PhoQ A20P	512	512	256
K. pneumoniae	141 CHX	WbaP D445E	2	64	16
K. pneumoniae	19 CHX	ΔmgrB	512	512	32-512
K. pneumoniae	20 CHX	MgrB deletion	256	256	256
K. pneumoniae	16 CHX	MgrB W20STOP	128	128	64
K. oxytoca	5490 CHX	MgrB deletion	8-128	32	8
A. baumannii	W1 CST_R	PmrB Duplication S17-F26	512	512	4
A. baumannii	UKA8 CST_RII	PmrB T235I	256	128-512	4
E. coli	NCTC 13846	*mcr-1*	2	1	0.5
E. coli	LEC001	PmrA R81C	8	≤0.0625	≤0.0625
E. coli	319238/UR	PmrB L10R	16	8	0.5
P. aeruginosa	GH12 CST	PmrB Δ115-121 amino acid	32-128	64	2
P. aeruginosa	Cas3 CST	PmrB R287Q	256	256	8
P. aeruginosa	NCTC 13437 CST	PmrB ΔD45	256	256	2

Table S4: MICs of fluspirilene and oxethazaine against the extended panel of Gram-negative species, and concentration at which each compound was used in combination with colistin to detect ARB activity.

		MIC (μM)		Potentiation concentration (μM)	
		oxe	flus	oxe	flus
K. pneumoniae	16 CST-R	>160	>160	20	20
K. pneumoniae	NCTC 13438 CST C	>160	>160	10	10
K. pneumoniae	NCTC 13438 CST D	>160	>160	10	10
K. pneumoniae	NCTC 13439 CST 1A	>160	>160	20	20
K. pneumoniae	NCTC 13439 CST 1C	>160	>160	20	20
K. pneumoniae	NCTC 13439 CST 2A	>160	>160	20	20
K. pneumoniae	NCTC 13439 CST 2B	>160	>160	20	20
K. pneumoniae	NCTC 13443 CST 2B	>160	>160	20	20
K. pneumoniae	NCTC 13443 CST	>160	>160	20	20
K. pneumoniae	MGH 78578 CST A	>160	>160	20	20
K. pneumoniae	MGH 78578 CST B	>160	>160	20	20
K. pneumoniae	m3 CST D	>160	>160	20	20
K. pneumoniae	m109 CST 1B	>160	>160	20	20
K. pneumoniae	m109 CST 2B	>160	>160	10	10
K. pneumoniae	NCTC 51851	>160	>160	20	20
K. pneumoniae	NCTC 49856	>160	>160	20	20
K. pneumoniae	NCTC 13368 CHX	>160	>160	10	10
K. pneumoniae	MGH 78578 CHX	>160	>160	10	10
K. pneumoniae	m109 CHX	>160	>160	20	20
K. pneumoniae	m3 CHX	>160	>160	20	20
K. pneumoniae	NCTC 13439 CHX	>160	>160	20	20
K. pneumoniae	NCTC 13443 CHX	>160	>160	20	20
K. pneumoniae	141 CHX	>160	>160	10	10
K. pneumoniae	19 CHX	>160	>160	20	20
K. pneumoniae	20 CHX	>160	>160	10	10
K. pneumoniae	16 CHX	>160	>160	20	20
K. oxytoca	5490 CHX	>160	>160	20	20
A. baumannii	W1 CST_R	20	20	5	10
A. baumannii	UKA8 CST_RII	20	20	5	10
E. coli	NCTC 13846	>160	>160	20	20
E. coli	LEC001	>160	80	20	20
E. coli	319238/UR	160	80	10	10
P. aeruginosa	GH12 CST	>160	>160	5	1.25
P. aeruginosa	Cas3 CST	>160	>160	5	2.5
P. aeruginosa	NCTC 13437 CST	>160	>160	10	10

Figure S1: A. Direct hits of compounds from the Prestwick library at 20 μM against all strains, coloured by therapeutic class. The blue dotted line represents the mean plus 3 standard deviations. Any compounds above this line are defined as hits. B. Makeup of the Prestwick library by therapeutic class. C. Hits by therapeutic class across all strains, with duplicate hits removed.

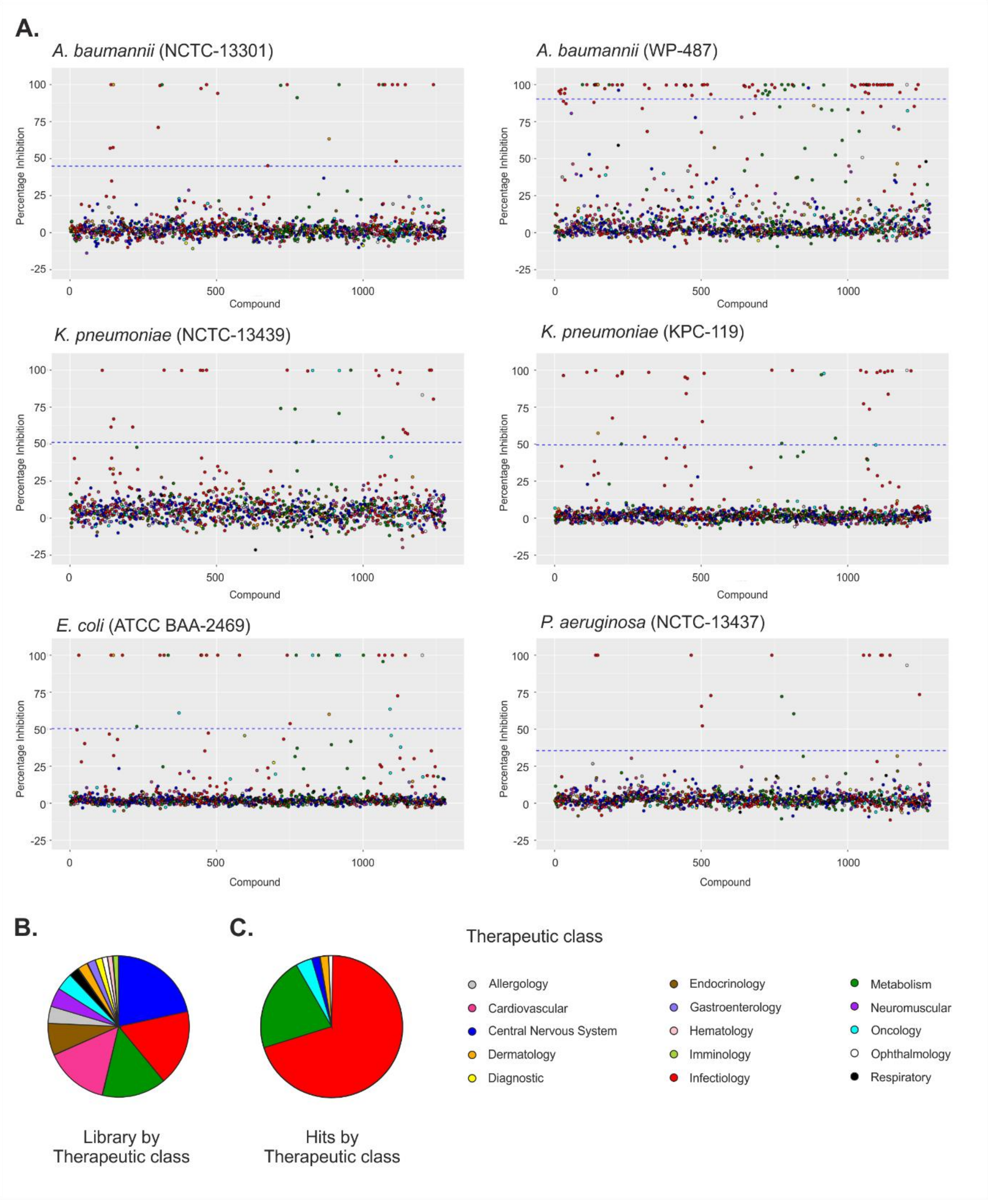

Figure S2: Direct hits for all strains at 7 μM.

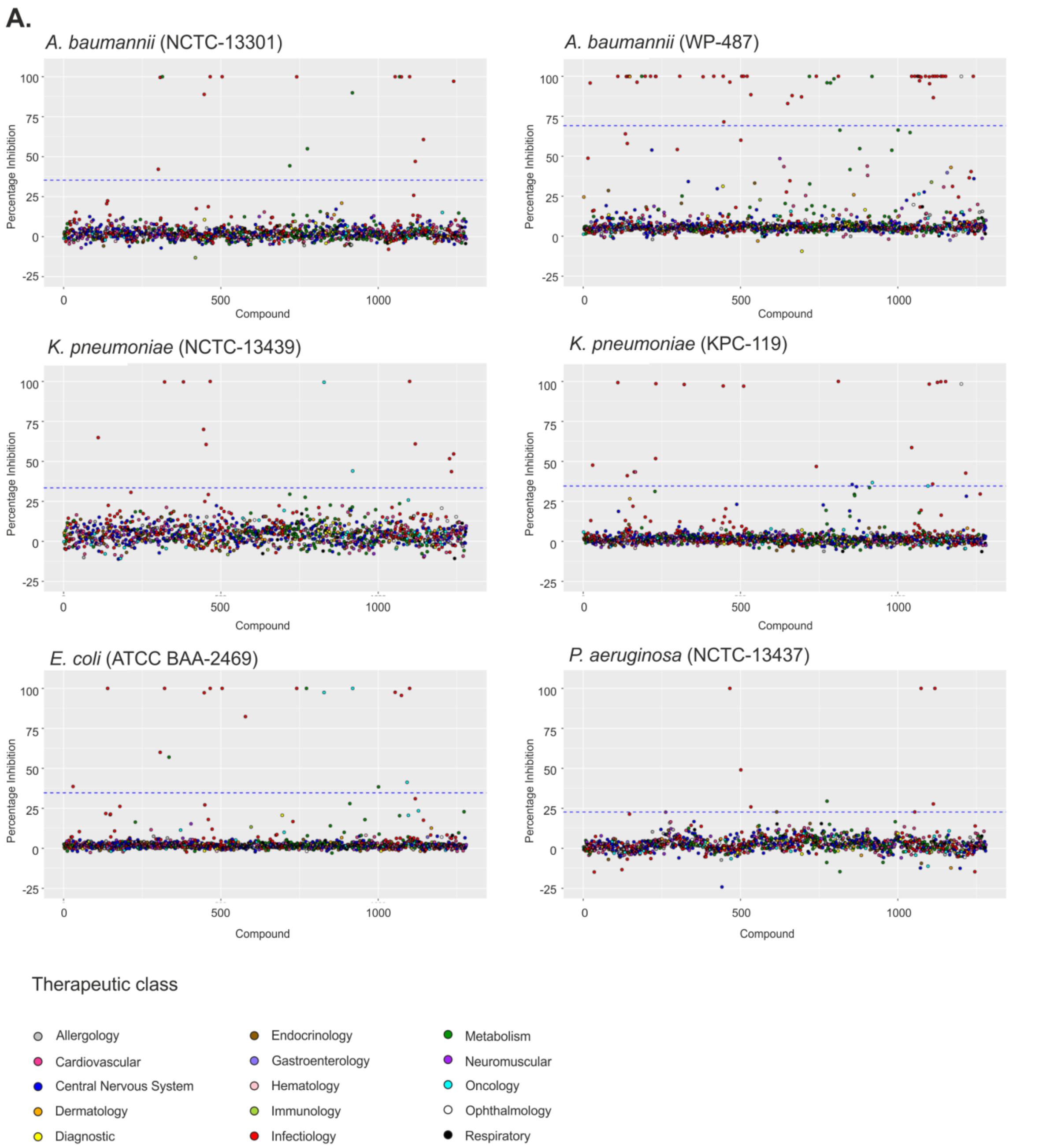

Figure S3: A. Antibiotic resistance breakers for *K. pneumoniae* in combination with all five antibiotics. For key, see Figure S1.

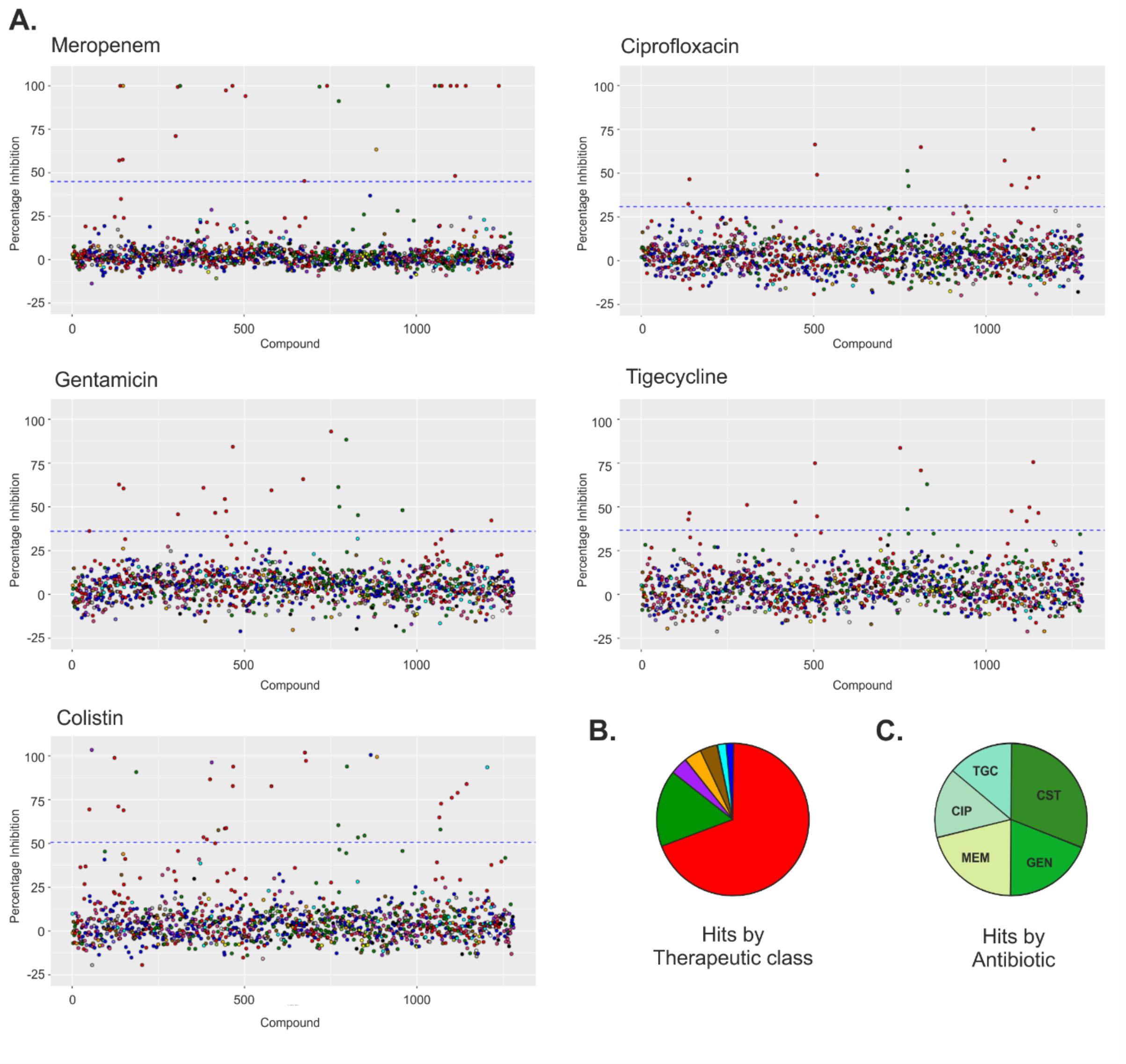

S4: ARB hits for *K. pneumoniae* at 7 μM.

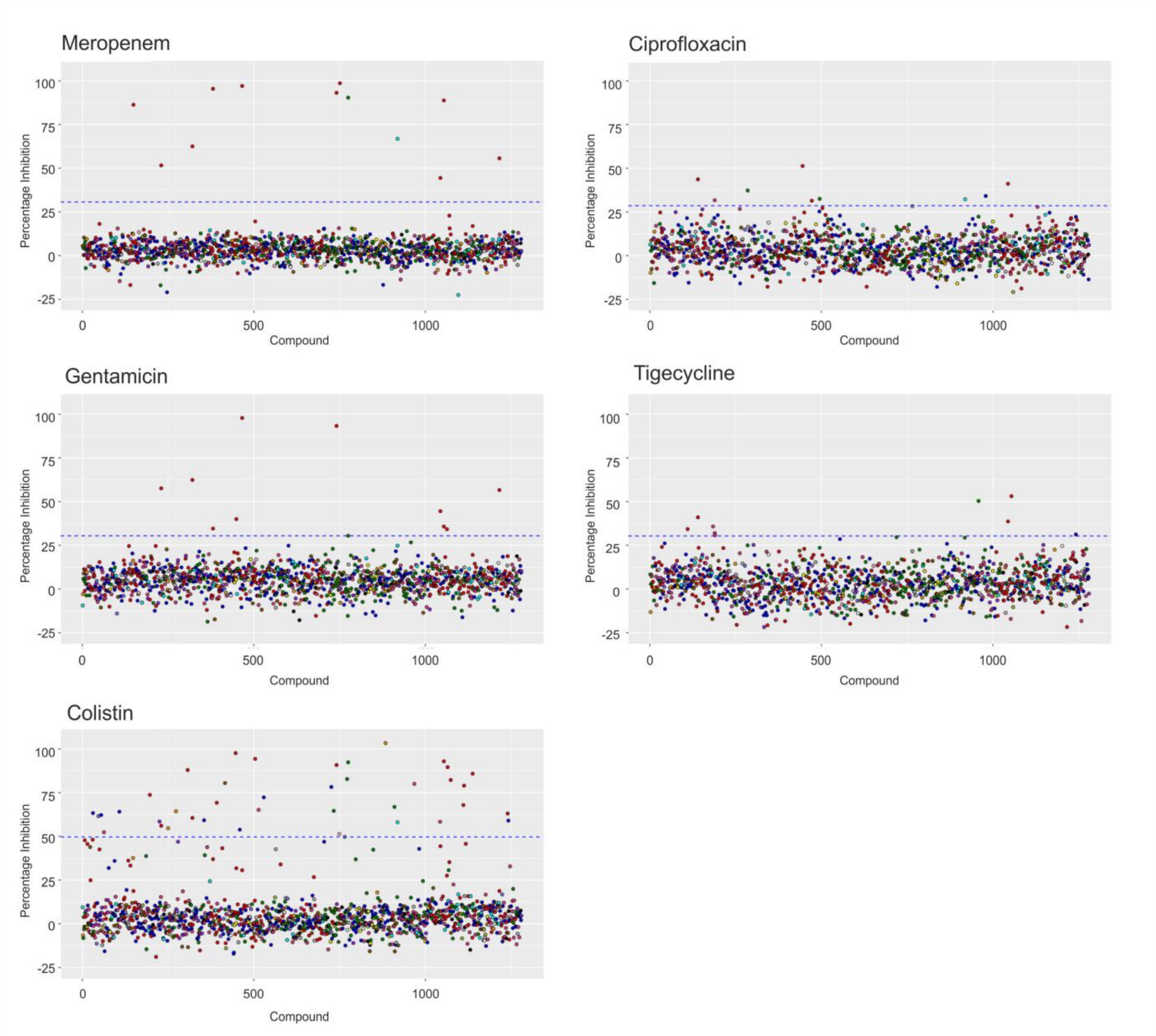

S5: ARB hits for *A. baumannii* NCTC-13301 at 20 μM and 7 μM.

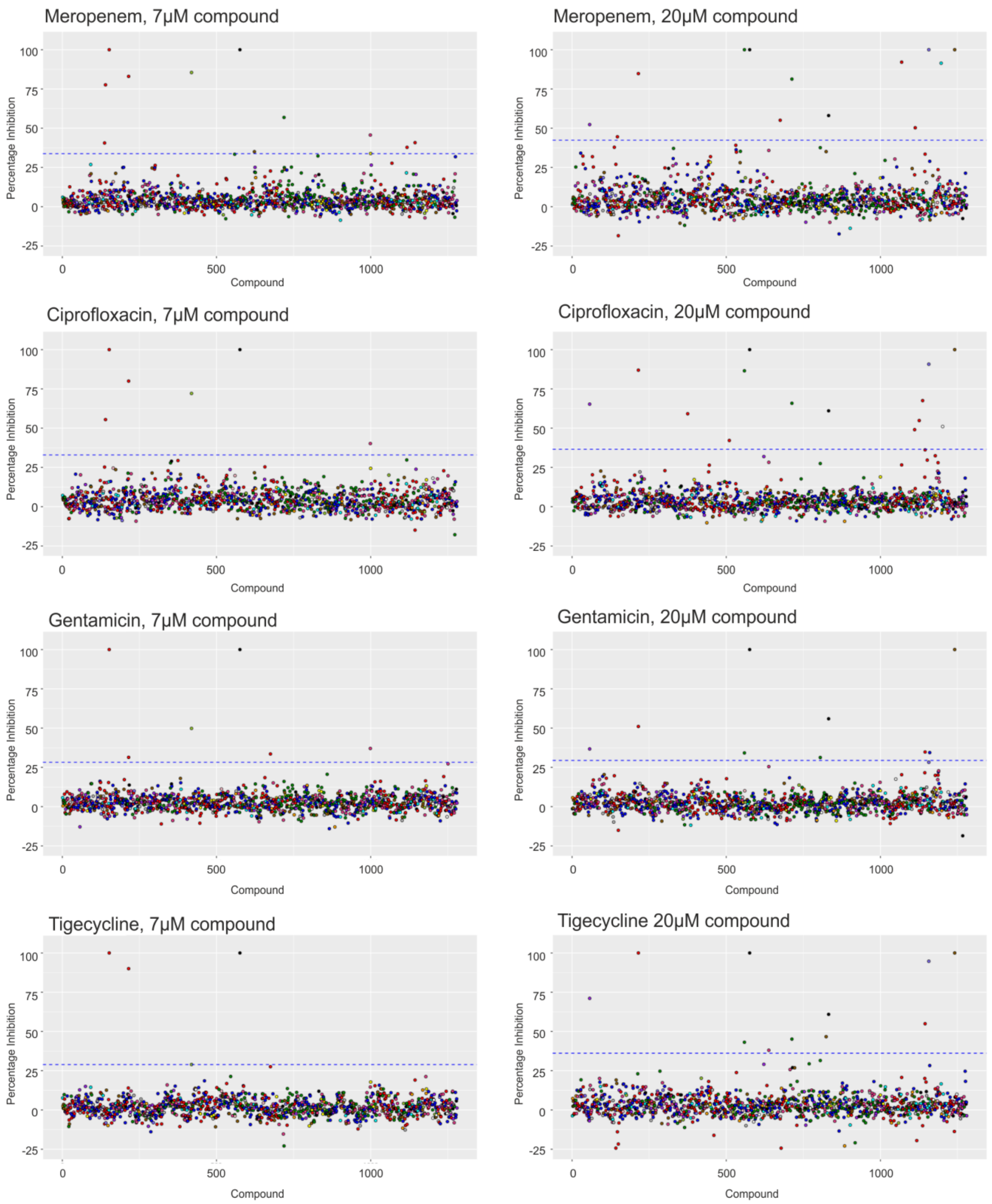

S6: ARB hits for *A. baumannii* WP-487 at 20 μM and 7 μM.

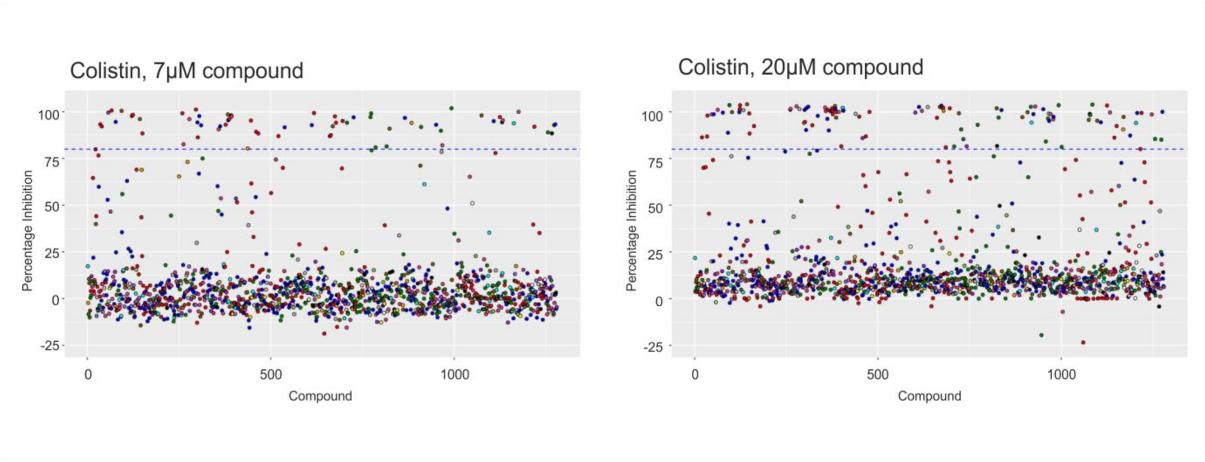

S7: ARB hits for *E. coli* ATCC BAA-2469 at 20 μM and 7 μM.

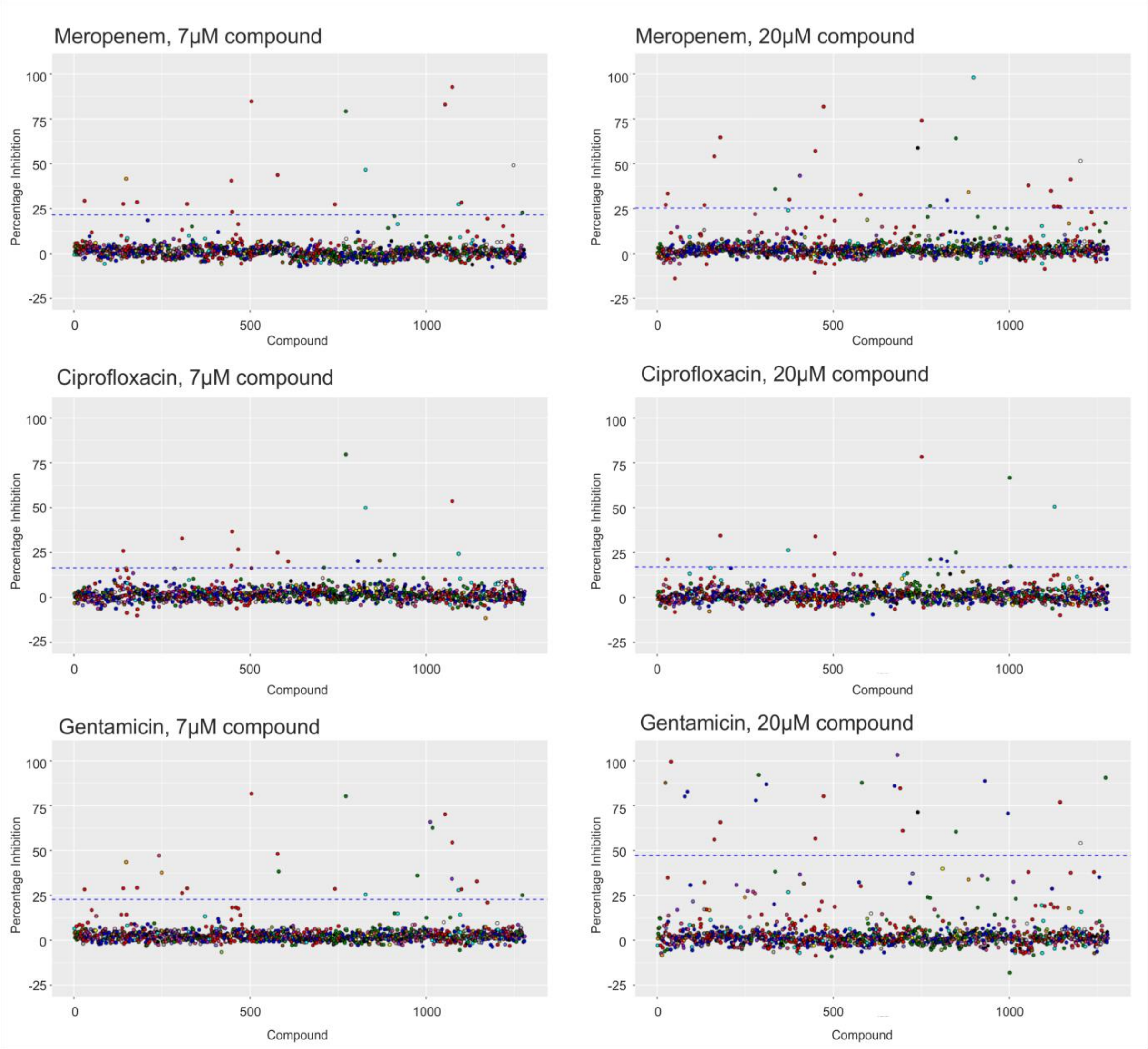

S8: ARB hits for *P. aeruginosa* NCTC-13437 at 20 µM and 7 µM.

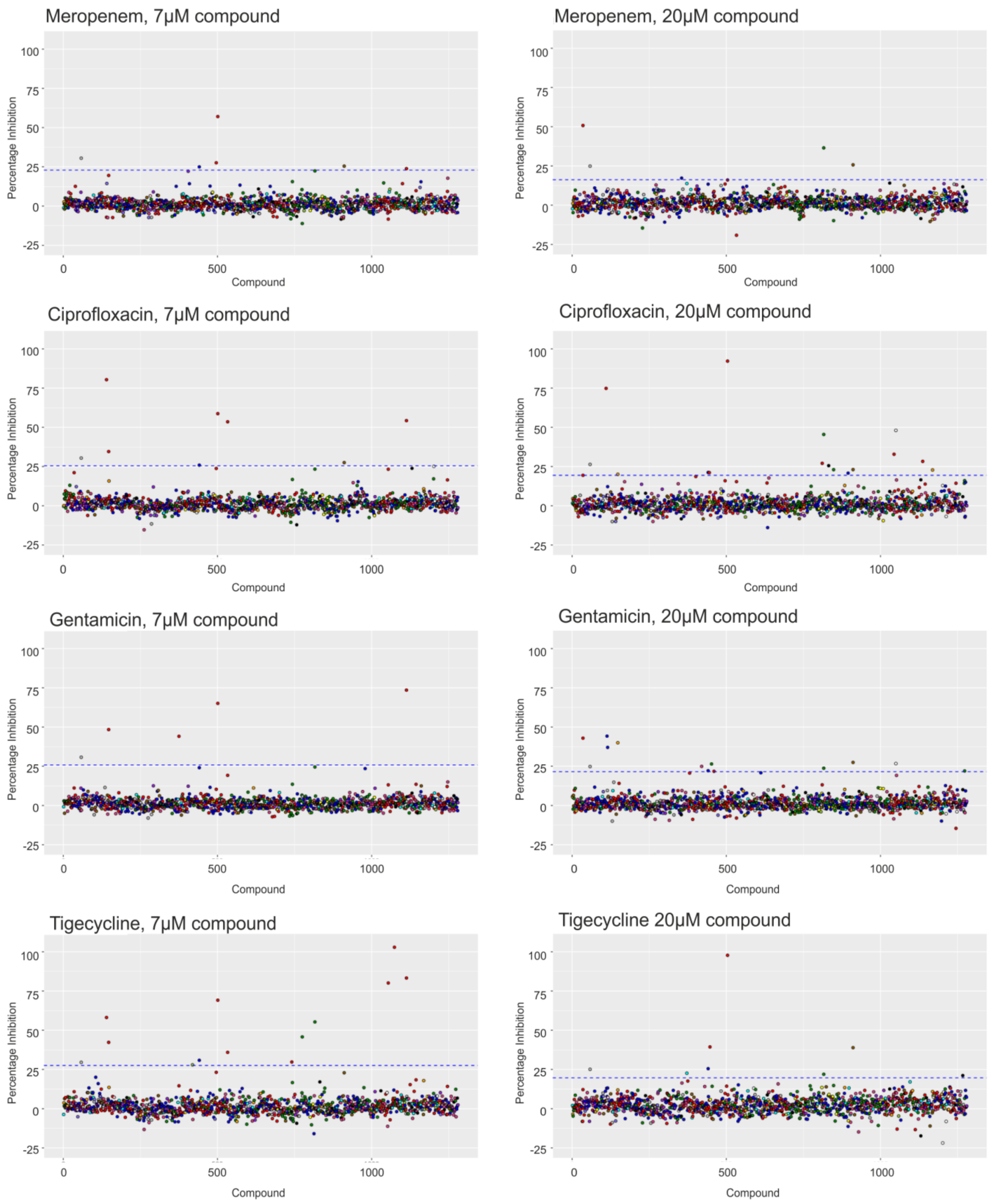

[1] Bock, L. J., Hind, C. K., Sutton, J. M., and Wand, M. E. (2018) Growth media and assay plate material can impact on the effectiveness of cationic biocides and antibiotics against different bacterial species, *Lett Appl Microbiol 66*, 368-377.

[2] Siles, S. A., Srinivasan, A., Pierce, C. G., Lopez-Ribot, J. L., and Ramasubramanian, A. K. (2013) High-throughput screening of a collection of known pharmacologically active small compounds for identification of Candida albicans biofilm inhibitors, *Antimicrobial agents and chemotherapy 57*, 3681-3687.

[3] Torres, N. S., Montelongo-Jauregui, D., Abercrombie, J. J., Srinivasan, A., Lopez-Ribot, J. L., Ramasubramanian, A. K., and Leung, K. P. (2018) Antimicrobial and Antibiofilm Activity of Synergistic Combinations of a Commercially Available Small Compound Library With Colistin Against Pseudomonas aeruginosa, *Front Microbiol 9*, 2541.

[4] Mohamed, Y. F., Abou-Shleib, H. M., Khalil, A. M., El-Guink, N. M., and El-Nakeeb, M. A. (2016) Membrane permeabilization of colistin toward pan-drug resistant Gram-negative isolates, *Braz J Microbiol 47*, 381-388.

[5] Zusman, O., Altunin, S., Koppel, F., Dishon Benattar, Y., Gedik, H., and Paul, M. (2017) Polymyxin monotherapy or in combination against carbapenem-resistant bacteria: systematic review and meta-analysis, *The Journal of antimicrobial chemotherapy 72*, 29-39.

[6] Henriksson, R., Holm, S., and Littbrand, B. (1990) Interactions between antibiotics and antineoplastic drugs on antibacterial activity in vitro, *Acta Oncol 29*, 43-46.

[7] Muggia, F. M. (2001) Liposomal encapsulated anthracyclines: new therapeutic horizons, *Curr Oncol Rep 3*, 156-162.

[8] Shen, J., Liu, H., Mu, C., Wolfram, J., Zhang, W., Kim, H. C., Zhu, G., Hu, Z., Ji, L. N., Liu, X., Ferrari, M., Mao, Z. W., and Shen, H. (2017) Multi-step encapsulation of chemotherapy and gene silencing agents in functionalized mesoporous silica nanoparticles, *Nanoscale 9*, 5329-5341.

[9] Hopp, D. C., Rabenstein, J., Rhea, J., Smith, C., Romari, K., Clarke, M., Francis, L., Irigoyen, M., Milanowski, D., Luche, M., Carr, G. J., and Mocek, U. (2008) Mutactimycin E, a new anthracycline antibiotic with gram-positive activity, *J Antibiot (Tokyo) 61*, 675-679.

[10] Andriollo, P., Hind, C. K., Picconi, P., Nahar, K. S., Jamshidi, S., Varsha, A., Clifford, M., Sutton, J. M., and Rahman, K. M. (2018) C8-Linked Pyrrolobenzodiazepine Monomers with Inverted Building Blocks Show Selective Activity against Multidrug Resistant Gram-Positive Bacteria, *ACS Infect Dis 4*, 158-174.

[11] Rahman, K. M., Rosado, H., Moreira, J. B., Feuerbaum, E. A., Fox, K. R., Stecher, E., Howard, P. W., Gregson, S. J., James, C. H., de la Fuente, M., Waldron, D. E., Thurston, D. E., and Taylor, P. W. (2012) Antistaphylococcal activity of DNA-interactive pyrrolobenzodiazepine (PBD) dimers and PBD-biaryl conjugates, *The Journal of antimicrobial chemotherapy 67*, 1683-1696.

[12] Furman, P. A., and Barry, D. W. (1988) Spectrum of antiviral activity and mechanism of action of zidovudine. An overview, *Am J Med 85*, 176-181.

[13] Shilaih, M., Angst, D. C., Marzel, A., Bonhoeffer, S., Gunthard, H. F., and Kouyos, R. D. (2018) Antibacterial effects of antiretrovirals, potential implications for microbiome studies in HIV, *Antivir Ther 23*, 91-94.

[14] Timofeev, V., Abramchik, Y., Zhukhlistova, N., Muravieva, T., Fateev, I., Esipov, R., and Kuranova, I. (2014) 3'-Azidothymidine in the active site of Escherichia coli thymidine phosphorylase: the peculiarity of the binding on the basis of X-ray study, *Acta Crystallogr D Biol Crystallogr 70*, 1155-1165.

[15] Doleans-Jordheim, A., Bergeron, E., Bereyziat, F., Ben-Larbi, S., Dumitrescu, O., Mazoyer, M. A., Morfin, F., Dumontet, C., Freney, J., and Jordheim, L. P. (2011) Zidovudine (AZT) has a

bactericidal effect on enterobacteria and induces genetic modifications in resistant strains, *Eur J Clin Microbiol Infect Dis 30*, 1249-1256.
[16] Loose, M., Naber, K. G., Hu, Y., Coates, A., and Wagenlehner, F. M. E. (2018) Serum bactericidal activity of colistin and azidothymidine combinations against mcr-1-positive colistin-resistant Escherichia coli, *Int J Antimicrob Agents 52*, 783-789.
[17] Hu, Y., Liu, Y., and Coates, A. (2019) Azidothymidine produces synergistic activity in combination with colistin against antibiotic-resistant Enterobacteriaceae, *Antimicrobial agents and chemotherapy 63*, e01630-01618.
[18] Wambaugh, M. A., Shakya, V. P. S., Lewis, A. J., Mulvey, M. A., and Brown, J. C. S. (2017) High-throughput identification and rational design of synergistic small-molecule pairs for combating and bypassing antibiotic resistance, *PLoS Biol 15*, e2001644.
[19] Ng, S. M. S., Sioson, J. S. P., Yap, J. M., Ng, F. M., Ching, H. S. V., Teo, J. W. P., Jureen, R., Hill, J., and Chia, C. S. B. (2018) Repurposing Zidovudine in combination with Tigecycline for treating carbapenem-resistant Enterobacteriaceae infections, *Eur J Clin Microbiol Infect Dis 37*, 141-148.
[20] Chang, A. E., Schneider, P. D., Sugarbaker, P. H., Simpson, C., Culnane, M., and Steinberg, S. M. (1987) A prospective randomized trial of regional versus systemic continuous 5-fluorodeoxyuridine chemotherapy in the treatment of colorectal liver metastases, *Ann Surg 206*, 685-693.
[21] Leichman, C. G. (1994) Prolonged infusion of fluorinated pyrimidines in gastrointestinal malignancies: a review of recent clinical trials, *Cancer Invest 12*, 166-175.
[22] Chionh, F., Lau, D., Yeung, Y., Price, T., and Tebbutt, N. (2017) Oral versus intravenous fluoropyrimidines for colorectal cancer, *Cochrane Database Syst Rev 7*, CD008398.
[23] Singlas, E., Taburet, A. M., Borsa Lebas, F., Parent de Curzon, O., Sobel, A., Chauveau, P., Viron, B., al Khayat, R., Poignet, J. L., Mignon, F., and et al. (1992) Didanosine pharmacokinetics in patients with normal and impaired renal function: influence of hemodialysis, *Antimicrob Agents Chemother 36*, 1519-1524.
[24] Geller, L. T., Barzily-Rokni, M., Danino, T., Jonas, O. H., Shental, N., Nejman, D., Gavert, N., Zwang, Y., Cooper, Z. A., Shee, K., Thaiss, C. A., Reuben, A., Livny, J., Avraham, R., Frederick, D. T., Ligorio, M., Chatman, K., Johnston, S. E., Mosher, C. M., Brandis, A., Fuks, G., Gurbatri, C., Gopalakrishnan, V., Kim, M., Hurd, M. W., Katz, M., Fleming, J., Maitra, A., Smith, D. A., Skalak, M., Bu, J., Michaud, M., Trauger, S. A., Barshack, I., Golan, T., Sandbank, J., Flaherty, K. T., Mandinova, A., Garrett, W. S., Thayer, S. P., Ferrone, C. R., Huttenhower, C., Bhatia, S. N., Gevers, D., Wargo, J. A., Golub, T. R., and Straussman, R. (2017) Potential role of intratumor bacteria in mediating tumor resistance to the chemotherapeutic drug gemcitabine, *Science 357*, 1156-1160.
[25] Moroni, G. N., Bogdanov, P. M., and Brinon, M. C. (2002) Synthesis and in vitro antibacterial activity of novel 5'-O-analog derivatives of zidovudine as potential prodrugs, *Nucleosides Nucleotides Nucleic Acids 21*, 231-241.
[26] Tsume, Y., Borras Bermejo, B., and Amidon, G. L. (2014) The dipeptide monoester prodrugs of floxuridine and gemcitabine-feasibility of orally administrable nucleoside analogs, *Pharmaceuticals (Basel) 7*, 169-191.
[27] Kruszewska, H., Zareba, T., and Tyski, S. (2004) Examination of antimicrobial activity of selected non-antibiotic drugs, *Acta Pol Pharm 61 Suppl*, 18-21.
[28] Kalaycı, S., Demirci, S., and Sahin, F. (2014) Antimicrobial Properties of Various Psychotropic Drugs Against Broad Range Microorganisms, *Current Psychopharmacology 3*, 195-202.
[29] Klitgaard, J. K., Skov, M. N., Kallipolitis, B. H., and Kolmos, H. J. (2008) Reversal of methicillin resistance in Staphylococcus aureus by thioridazine, *The Journal of antimicrobial chemotherapy 62*, 1215-1221.
[30] Poulsen, M. O., Jacobsen, K., Thorsing, M., Kristensen, N. R., Clasen, J., Lillebaek, E. M., Skov, M. N., Kallipolitis, B. H., Kolmos, H. J., and Klitgaard, J. K. (2013) Thioridazine potentiates the

effect of a beta-lactam antibiotic against Staphylococcus aureus independently of mecA expression, *Res Microbiol 164*, 181-188.
[31] Amaral, L., Kristiansen, J. E., Abebe, L. S., and Millett, W. (1996) Inhibition of the respiration of multi-drug resistant clinical isolates of Mycobacterium tuberculosis by thioridazine: potential use for initial therapy of freshly diagnosed tuberculosis, *The Journal of antimicrobial chemotherapy 38*, 1049-1053.
[32] Nzakizwanayo, J., Scavone, P., Jamshidi, S., Hawthorne, J. A., Pelling, H., Dedi, C., Salvage, J. P., Hind, C. K., Guppy, F. M., Barnes, L. M., Patel, B. A., Rahman, K. M., Sutton, M. J., and Jones, B. V. (2017) Fluoxetine and thioridazine inhibit efflux and attenuate crystalline biofilm formation by Proteus mirabilis, *Sci Rep 7*, 12222.
[33] Cheng, Y. S., Sun, W., Xu, M., Shen, M., Khraiwesh, M., Sciotti, R. J., and Zheng, W. (2018) Repurposing Screen Identifies Unconventional Drugs With Activity Against Multidrug Resistant Acinetobacter baumannii, *Front Cell Infect Microbiol 8*, 438.
[34] Cortes Cabrera, A., Lucena-Agell, D., Redondo-Horcajo, M., Barasoain, I., Diaz, J. F., Fasching, B., and Petrone, P. M. (2016) Aggregated Compound Biological Signatures Facilitate Phenotypic Drug Discovery and Target Elucidation, *ACS Chem Biol 11*, 3024-3034.
[35] Parakh, R. K., and Patil, N. S. (2018) Anaesthetic antacids: a review of its pharmacological properties and therapeutic efficacy, *International Journal of Research in Medical Sciences 6*, 383-393.
[36] Sims, K. R., Liu, Y., Hwang, G., Jung, H. I., Koo, H., and Benoit, D. S. W. (2018) Enhanced design and formulation of nanoparticles for anti-biofilm drug delivery, *Nanoscale 11*, 219-236.
[37] Liu, M., Landuyt, B., Klaassen, H., Geldhof, P., and Luyten, W. (2019) Screening of a drug repurposing library with a nematode motility assay identifies promising anthelmintic hits against Cooperia oncophora and other ruminant parasites, *Vet Parasitol 265*, 15-18.
[38] Niu, H., Yee, R., Cui, P., Tian, L., Zhang, S., Shi, W., Sullivan, D., Zhu, B., Zhang, W., and Zhang, Y. (2017) Identification of Agents Active against Methicillin-Resistant Staphylococcus aureus USA300 from a Clinical Compound Library, *Pathogens 6*.
[39] Gao, L., Sun, Y., He, C., Zeng, T., and Li, M. (2018) Synergy between Pyrvinium Pamoate and Azoles against Exophiala dermatitidis, *Antimicrobial agents and chemotherapy 62*.
[40] Holbrook, S. Y., Garzan, A., Dennis, E. K., Shrestha, S. K., and Garneau-Tsodikova, S. (2017) Repurposing antipsychotic drugs into antifungal agents: Synergistic combinations of azoles and bromperidol derivatives in the treatment of various fungal infections, *European journal of medicinal chemistry 139*, 12-21.
[41] Yssel, A., Vanderleyden, J., and Steenackers, H. (2017) Repurposing of nucleoside-and nucleobase-derivative drugs as antibiotics and biofilm inhibitors, *Journal of Antimicrobial Chemotherapy 72*, 2156-2170.
